# A 16S rDNA sequencing-based analysis of airway microecology in patients with an acute exacerbation of chronic obstructive pulmonary disease: A cross-sectional study in Inner Mongolia, China

**DOI:** 10.3389/fmed.2022.946238

**Published:** 2022-10-13

**Authors:** Shu-fen Zhu, Xin-xin Wu, Yan Guo, Peng-fei Li, Jing-ran Wang, Miao Liu, Cheng-wen Luo, Xiang-zhen Yuan, Shao-wei Li

**Affiliations:** ^1^Physical Examination Center, The Affiliated Hospital of Inner Mongolia Medical University, Hohhot, China; ^2^Inner Mongolia Medical University, Hohhot, China; ^3^Department of Pathology, Inner Mongolia People’s Hospital, Hohhot, China; ^4^Department of Orthopaedics, Inner Mongolia People’s Hospital, Hohhot, China; ^5^Evidence-Based Medicine Center, Taizhou Hospital of Zhejiang Province, Wenzhou Medical University, Linhai, China; ^6^Key Laboratory of Minimally Invasive Techniques & Rapid Rehabilitation of Digestive System Tumor of Zhejiang Province, Taizhou Hospital Affiliated to Wenzhou Medical University, Linhai, China; ^7^Department of Gastroenterology, Taizhou Hospital of Zhejiang Province Affiliated to Wenzhou Medical University, Linhai, China; ^8^Institute of Digestive Disease, Taizhou Hospital of Zhejiang Province Affiliated to Wenzhou Medical University, Linhai, China

**Keywords:** chronic obstructive pulmonary disease, acute exacerbation, airway microecology, 16s rDNA sequencing, sputum samples

## Abstract

**Aim:**

To study the microecological characteristics of the airway and similarities and differences between healthy people and patients with the acute exacerbation of chronic obstructive pulmonary disease (AECOPD) in Inner Mongolia, and analyze the correlation between the characteristics of the airway microecological structure and clinical indicators of AECOPD patients.

**Methods:**

Sputum samples from 36 healthy volunteers and 34 patients with AECOPD were detected by 16S rDNA high-throughput sequencing, and the airway microecological characteristics of healthy people and AECOPD patients were revealed by an alpha diversity analysis, beta diversity analysis, and LefSe difference analysis.

**Results:**

There were differences in the airway microecological structure between healthy people and AECOPD patients in Inner Mongolia. The airway microbiota composition of AECOPD patients showed an increase in the abundance of common pathogens and a decrease in the abundance of commensal bacteria, and the airway microbial diversity in AECOPD patients was lower than that in healthy people. Long-term use of inhaled glucocorticoid + long-acting β2 agonist mixture (ICS + LABA), procalcitonin (PCT), blood monocyte count (MONO), hemoglobin (HGB), D-dimer (D-D), and body temperature were negatively correlated with the alpha diversity of the airway micro-ecosystem.

**Conclusion:**

The airway microecological composition of the AECOPD population in Inner Mongolia was different from that of the healthy population, and the airway microecological diversity was lower than that of the healthy population. The long-term use of ICS + LABA preparation by patients with AECOPD leads to lower alpha diversity. Alpha diversity was negatively correlated with inflammatory markers (PCT, MONO, D-dimer, body temperature) and HGB in AECOPD patients.

## Introduction

Chronic pulmonary disease obstructive (COPD) is a common chronic respiratory disease characterized by continuous airflow restriction (1). COPD patients suffer from the sudden exacerbation of symptoms and even respiratory failure, termed the acute exacerbation of chronic obstructive pulmonary disease (AECOPD). This is the main reason for increased mortality in patients with COPD. The pathogenesis of AECOPD is complex, involving infection, air pollution, high reactivity of the respiratory tract, and many other factors (2). In the past, because of the limitation of traditional sputum culture technology, the multiplication of one or several types of pathogenic bacteria was believed to be the main reason for AECOPD. However, with the emergence of a new generation of sequencing technology, the microecological structure of the airway was found to be closely associated with the onset of AECOPD and deterioration. After drug treatment, the microecology of the airway also changes in individual patients, which affects the course of the disease. In this background, we applied 16SrDNA high-throughput sequencing to analyze airway the microecology of healthy Han people and patients with AECOPD in Inner Mongolia in order to provide airway microecological data for these populations and to explore the correlation between the airway microecology and clinical indicators in AECOPD patients.

## Materials and methods

### Subjects and observations

The present study included individuals with AECOPD who were treated as inpatients in the Department of Respiratory and Critical Care Medicine, Affiliated Hospital of Inner Mongolia Medical University from January 2021 to December 2021. Healthy volunteers were recruited from individuals who underwent a physical examination at the same time. A total of 34 hospitalized AECOPD volunteers and 36 healthy volunteers were included in this study. The inclusion criteria for the AECOPD group were as follows: a diagnosis of AECOPD in line with the 2019 Global Initiative for Chronic Obstructive Pulmonary Disease (3), no history of antibiotic use in the 3 months before admission, and living in Inner Mongolia for a long time. Patients with bronchial asthma, pulmonary tuberculosis, interstitial lung disease, other chronic lung diseases, acute pulmonary edema, acute pulmonary embolism, acute heart failure, arrhythmia, other cardiopulmonary diseases, tumors, and autoimmune diseases were excluded from the study. The inclusion criteria for the healthy group were as follows: no chronic respiratory disease (e.g., COPD, pulmonary fibrosis, pulmonary tuberculosis, lung tumor, bronchial asthma, bronchiectasis, pulmonary sarcoidosis, etc.); no history of diseases affecting the immune system (e.g., blood system and autoimmune disease), age > 18 years; and living in Inner Mongolia for a long time. Patients who were unable to cooperate with sputum collection had a history of infection within the previous 3 months, or had a history of antibiotic use in the previous 3 months were excluded from the study. After obtaining their informed consent, sputum samples were collected from all volunteers and basic information, such as age, sex, and smoking status, was provided. Clinical case data were provided by volunteers in the AECOPD group. In addition, peripheral blood was extracted from all AECOPD patients before treatment on the day of admission and sent to the clinical laboratory of our hospital for a complete analysis of blood routine parameters, an evaluation of high-sensitivity C-reactive protein (hs-CRP), procalcitonin, a liver function test, sputum culture, and other analyses. All healthy volunteers came from the physical examination center of the same hospital. The physical examination items included lung function, chest CT, blood routine, liver function, kidney function, etc.

### Methods

#### Collection of sputum samples

All subjects were repeatedly rinsed with normal saline to remove nasal and mouth secretions before sputum collection, and standards of operation were strictly observed during sputum collection in order to avoid contamination as far as possible. For objects with a large amount of sputum, the natural expectoration method was used to collect sputum samples, and the sputum from the deep part of the trachea was forcibly expectorated into sterile containers. For volunteers with no or low sputum volume, sputum samples were collected according to the improved Pin method (4): 3–5% gradient hypertonic saline air compression atomization was used for induction, the atomization time was 15 min, and the sputum was retained within 20 min. All sputum specimens were examined by sputum smear microscopy. Specimens with < 10 squamous cells/low magnification field and > 25 white blood cells/low magnification field were considered to be qualified sputum samples. Qualified sputum samples of at least 2 ml were collected in sterile cryopreservation tubes and frozen at -80^°^C within 2 h. Finally, the samples were uniformly sent to Hangzhou Lianchuan Biotechnology Co., Ltd., for a follow-up experimental analysis.

#### Bacterial deoxyribonucleic acid extraction, library construction, and a sequencing analysis

First, a Pathogenic Microbiome deoxyribonucleic acid (DNA) Kit (CWBIO) was used for the manual extraction of bacterial DNA from sputum samples. A PCR kit (New England Biolabs) was used with the341F (5’-CCTACGGGNGGCWGCAG-3’); 805R (5’-GACTACHVGGGTATCTAATCC-3’) primer to amplify the V3-V4 specific fragment of the 16S rDNA gene. PCR products were purified by AMPure XT Beads (Beckman Coulter Genomics, USA), quantified by Qubit (Invitrogen, USA), and then were recovered by 2% agarose gel electrophoresis. The amplicon pools were prepared for sequencing and the size and quantity of the amplicon library were assessed on Agilent 2100 Bioanalyzer (Agilent, USA) and with the Library Quantification Kit for Illumina (Kapa Biosciences, Woburn, MA, USA), respectively. The libraries were sequenced on NovaSeq 6000 SP platform. Samples were sequenced on an Illumina NovaSeq platform paired-end reads were assigned to samples based on their unique barcode and truncated by cutting off the barcode and primer sequence. Paired-end reads were merged using FLASH (http://ccb.jhu.edu/software/FLASH/). Quality filtering on the raw reads was performed under specific filtering conditions to obtain high-quality clean tags according to the fqtrim (v0.94). Chimeric sequences were filtered using Vsearch software (v2.3.4). After dereplication using DADA2, we obtained the feature table and the feature sequence. According to the sequence of the ASV file using the SILVA (Release 138) database to NT-16s annotation database for species, the confidence threshold of the comments was determined to be 0.7.

### Statistical analysis

The SPSS 22.0 software program was used for the basic statistical analysis of the data, and the T or T’ test was used to measure data with normal distribution. Non-normal data were tested by a non-parametric Mann–Whitney U test. Qualitative data were compared using Pearson’s chi-squared test. Spearman’s rank correlation analysis was used to analyze the correlation between variables, and *p*-values of < 0.05 were considered to indicate statistical significance. A bioinformatics analysis was performed using the R software program (V2.5.4). An alpha diversity analysis and beta diversity analysis were performed based on the obtained ASV (feature) feature sequence and ASV (feature) abundance table. In the alpha diversity analysis, QIIME2 (2019.7) was used to calculate Observed-outs, Shannon, Chao1, and other indices, while the R software program was used to draw dilution curves and analyze differences between groups. Beta diversity was calculated by QIIME2 and plotted by the R software program. LEfSe was used to analyze the species differences between the groups. The threshold value of the linear discriminant analysis effect size (LEfSe) analysis in this project was set as LDA value > 4, *p* < 0.05.

We used the *t*-test to calculate the difference between two independent groups, assuming the effect size level of 0.8, the significance level of 0.05, and the power of 0.80. The target sample size was 54 participants. We allowed for a 20% participant dropout (reluctance to participate) and selected 68 participants as a conservative sample size. The sample size was calculated using the software G.Power 3.1.9.6.

## Results

### Basic information

A total of 36 healthy volunteers were included in this study (male, *n* = 17; female, *n* = 19; smokers, *n* = 15; non-smokers, *n* = 21; Smoking index, 425.21 ± 417.68; average age, 68.51 ± 11.28 years). The AECOPD group included 34 AECOPD patients (male, *n* = 20; female, *n* = 14; smokers, *n* = 18 smokers; non-smokers, *n* = 16; Smoking index, 447.83 ± 471.81; average age, 72.03 ± 7.72 years). There were no significant differences between the two groups in age, sex, or smoking habits (*p* > 0.05). The AECOPD included in this study were all severe patients who required hospitalization according to their comprehensive evaluation of clinical manifestations and laboratory tests. The clinical data of the AECOPD patients included: disease course, test results on admission (hs-CRP, PCT, white blood cell count [WBC], neutrophil count [NEUT], neutrophil percentage [NEUT%], eosinophil count [EO], eosinophil ratio [EO%], lymphocyte count [LYM], lymphocyte ratio [LYM%], monocyte count [MONO], monocyte ratio [MONO%], basophil count [BASO%], basophil ratio [BASO%], hemoglobin [HGB], platelet count [PLT], prealbumin [PA], albumin [ALB], D-dimer [D-D]), body temperature, length of hospitalization, or long-term use of ICS + LABA mixed preparations (Table 1).

**TABLE 1:** Clinical information of acute exacerbation of chronic obstructive pulmonary disease (AECOPD) volunteers.

Index	AECOPD patient (*n* = 34)
Course of disease (year, *x* ± *s*)	11.79 ± 9.09
WBC (× 10^9^/L, *x* ± *s*)	7.91 ± 2.91
NEUT (× 10^9^/L, *x* ± *s*)	5.70 ± 2.64
NEUT% (%, *x* ± *s*)	70.91 ± 11.43
LYM (× 10^9^/L, *x* ± *s*)	1.39 ± 0.72
LYM% (%, *x* ± *s*)	18.36 ± 8.34
MONO (× 10^9^/L, *x* ± *s*)	0.53 ± 0.20
MONO% (%, *x* ± *s*)	7.48 ± 4.48
EO (× 10^9^/L, *x* ± *s*)	0.46 ± 0.99
EO% (%, *x* ± *s*)	3.77 ± 5.48
BASO (× 10^9^/L, *x* ± *s*)	0.04 ± 0.03
BASO% (%, *x* ± *s*)	0.46 ± 0.35
HGB (g/L)	150.14 ± 26.10
PLT (× 10^9^/L, *x* ± *s*)	197.15 ± 93.56
hs-CRP (mg/L)	14.79 ± 20.28
PCT (ng/mL)	0.54 ± 2.06
PA (mg/dL)	16.62 ± 7.00
ALB (g/L)	36.09 ± 7.73
D-D (μg/mL)	0.82 ± 0.84
Body temperature (^°^C)	36.6 ± 0.66
Length of stay (day)	8.88 ± 2.67
Use ICS + LABA (same)	17

### Airway microecological structure

**FIGURE 1:**
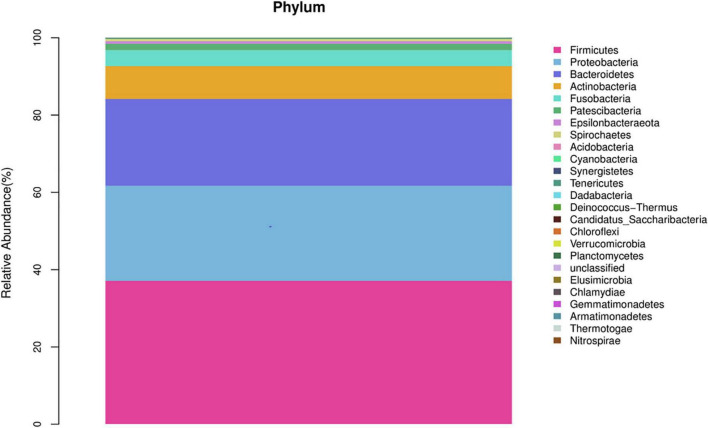
Histogram of microecological species distribution in healthy airways at the phylum level.

**FIGURE 2:**
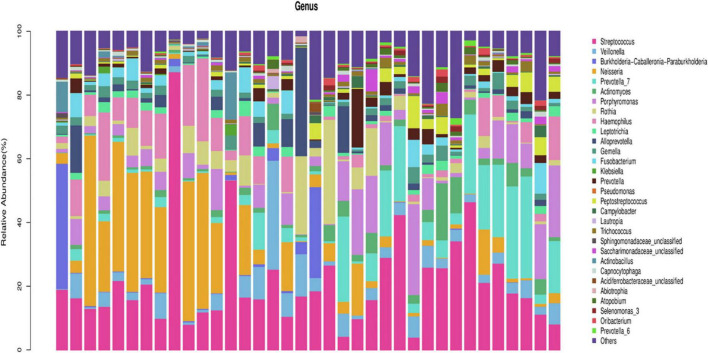
Histogram of microecological species distribution in healthy airways at the genus level.

In the healthy group, at the phylum level, Firmicutes, Proteobacteria, Bacteroidetes, Actinobacteria, and Fusobacteria constituted approximately 96.76% of the sequence in the community (Figure 1). At the genus level, the top 10 genera in the airway microecology of the healthy group were as follows Streptococcus, Neisseria, Prevotella-7, Porphyromonas, Haemophilus, Veillonella, Rothia, Alloprevotella, Actinomyces, and Fusobacterium, and these constituted approximately 73.63% of the sequences in the community (Figure 2).

**FIGURE 3:**
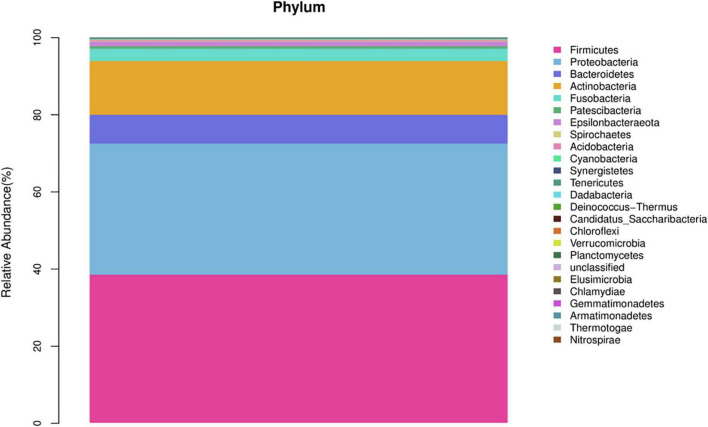
Composition of airway microbiota in the acute exacerbation of chronic obstructive pulmonary disease (AECOPD) group.

**FIGURE 4:**
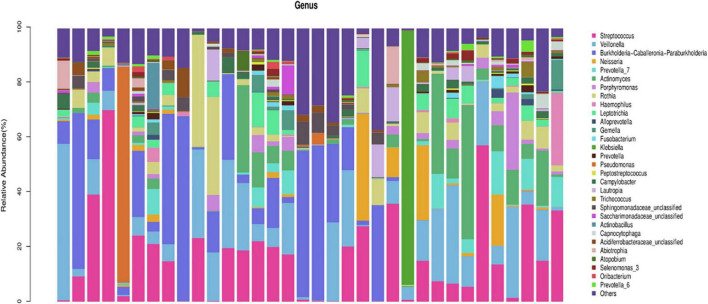
Composition of horizontal bacterial flora in the airway microecology of the acute exacerbation of chronic obstructive pulmonary disease (AECOPD) group.

In the AECOPD group at the phylum level, Firmicutes, Proteobacteria, Actinobacteria, Bacteroidetesa, and Fusobacteria constituted about 97.03% of the sequence in the community (Figure 3). The top 10 members of the genus were Streptococcus, Burkholderia, Veillonella, Actinomyces, Rothia, Neisseria, Prevotella-7, Klebsiella, Leptotrichia, and Pseudomonas; these constitute approximately 72.21% of the sequences in the community. Among the 34 AECOPD patients, most had a similar airway microecological composition, which maintained a dynamic balance among Pseudomonas, Burkholderia, and Veillonella. However, in some samples, Klebsiella and Pseudomonas were dominant (Figure 4).

### LefSe difference analysis

**FIGURE 5:**
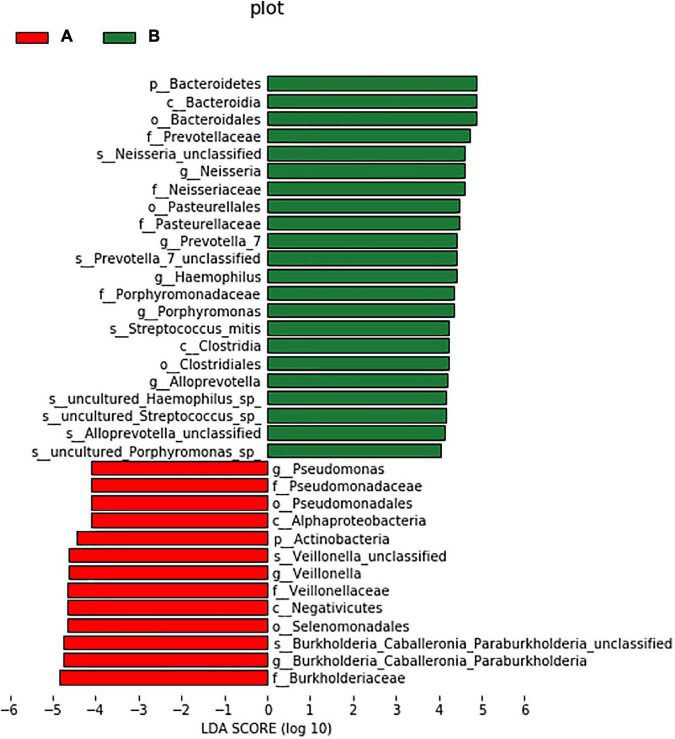
Histogram of the LDA value distribution of significantly different species.

**FIGURE 6:**
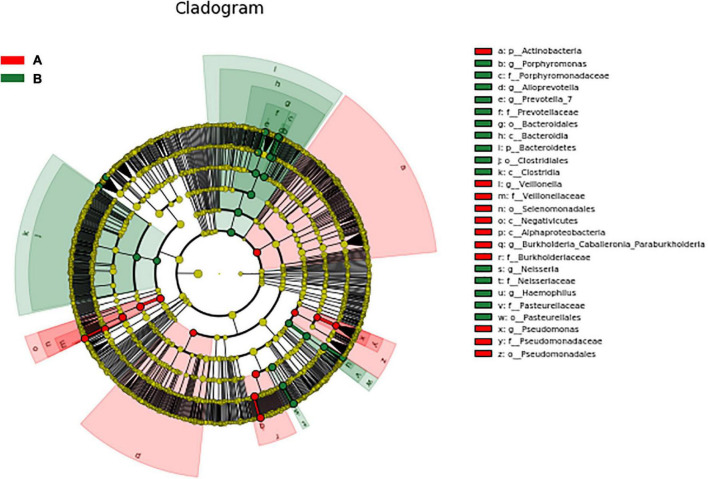
Evolutionary clade of significantly different species.

LEfSe was used to further search for bacterial communities with significant differences between groups. There were 35 microorganisms with LDA values of > 4 at different classification levels (A: AECOPD group, B: healthy group). The relative abundance of Bacteroidetes in the healthy group increased at the phylum level. At the genus level, the relative abundance of Pseudomonas, Veillonella, and Burkholderia in the AECOPD group was increased in comparison to the healthy group. The relative abundance of Neisseria, Prevotella-7, Haemophilus, Alloprevotella, and Porphyromonas was decreased in comparison to the healthy group (Figure 5). Figure 6 shows an evolutionary clade demonstrating the difference in the abundance of biomarkers between the two groups using two different colors.

### Alpha diversity

**FIGURE 7:**
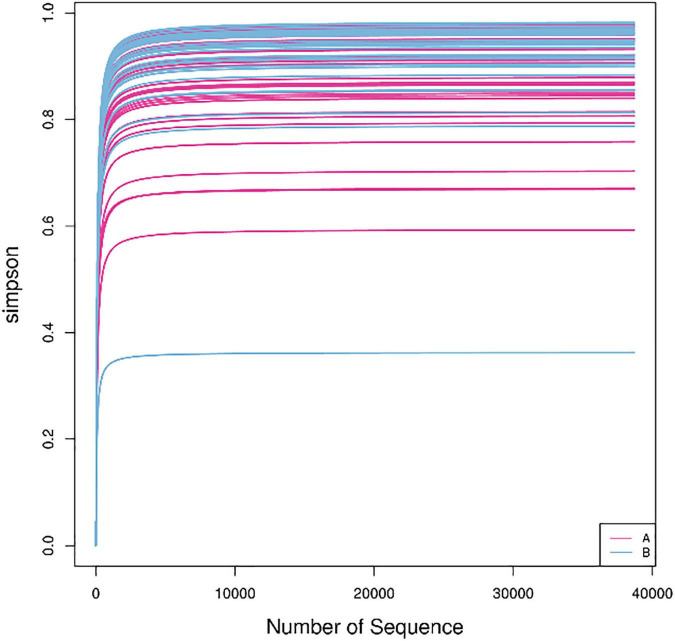
Dilution curve.

**FIGURE 8:**
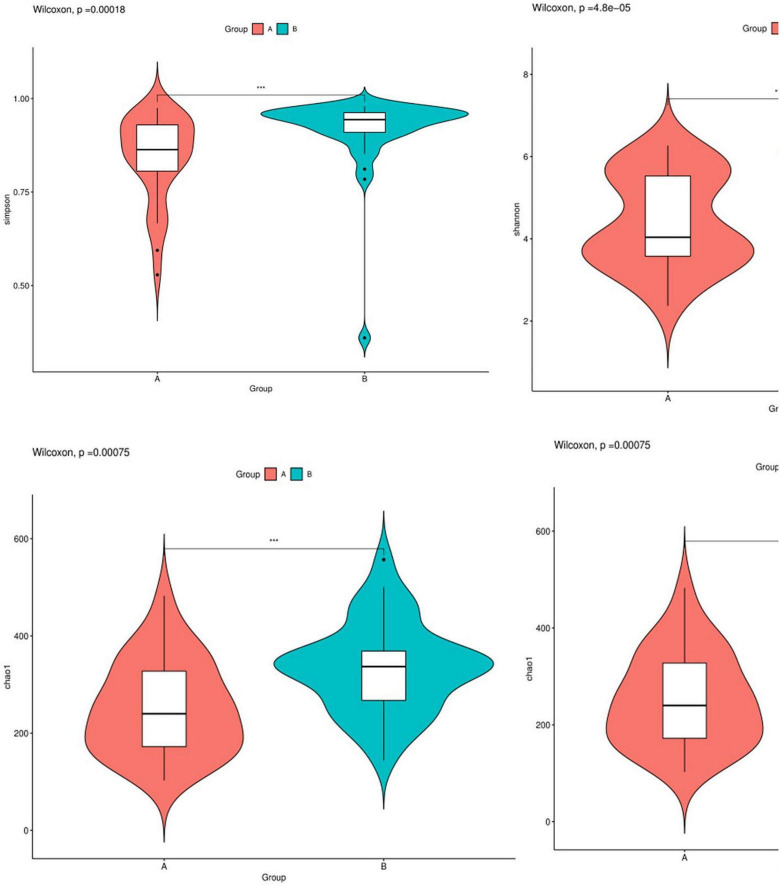
Difference analysis of alpha diversity index between groups.

In this study, the complexity of the diversity of the microbial community was analyzed by the alpha diversity index (Observed-outs, Shannon, Simpson, Chao1). The dilution curve drawn by the Simpson index value as an ordinate tends to be flat, indicating that the current sequencing depth covers most microorganisms. The curve also indirectly reflected that the alpha diversity of airway bacteria in the healthy group was higher than that in the AECOPD group (Figure 7). Through the analysis of significant differences between groups, it was found that the alpha diversity of the airway bacteria in the healthy group was higher than that in the AECOPD group (Observed-outs, Shannon, Simpson, and Chao1 comparisons were all *p* < 0.05) (Figure 8).

### Beta diversity analysis

**FIGURE 9:**
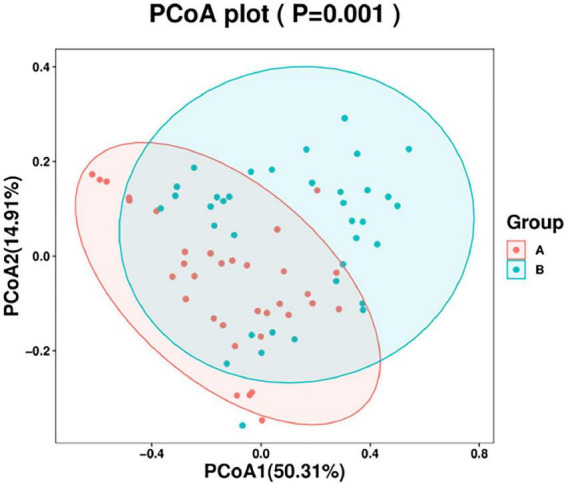
Principal coordinate (PcoA) analysis chart.

In this study, a principal coordinate (PCoA) analysis based on the Weighted UniFrac distance was performed (ANOSIM, *p* = 0.001). The PC1 contribution rate was 50.31%, and the PC2 contribution rate was 14.91%, indicating differences in the airway microecological structure between healthy individuals and patients with AECOPD (Figure 9).

### Relationship between acute plus recombinant alpha diversity index and clinically relevant indicators in chronic obstructive pulmonary disease

**FIGURE 10:**
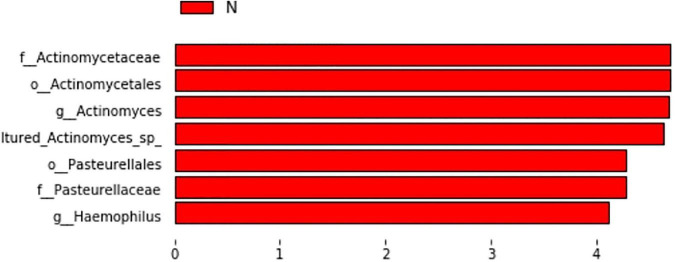
Histogram of LDA value distribution of significantly different species in subgroups.

We paid special attention to whether ICS + LABA affected the microecological structure of the airway, and the results showed that in the AECOPD group, ICS + LABA reduced alpha diversity (Observed-outs,*, Shannon*, Simpson*) (Table 2). LefSe was used to analyze the difference in flora between the two subgroups (Y: ICS + LABA, N: ICS + LABA), and it was found that the relative abundance of Actinomyces and Haemophilus was higher without ICS + LABA preparation (Figure 10).

**TABLE 2:** Differences in alpha diversity of airway microflora and ICS + LABA in the acute exacerbation of chronic obstructive pulmonary disease (AECOPD) group [median (interquartile range)].

	ICS + LABA		*P*
	
	Exist	Inexistence	
Observed-OTUs	175.00 (139.00)	265.00 (148.50)	0.036*
Shannon	3.85 (1.00)	5.38 (2.09)	0.026*
Simpson	0.84 (0.11)	0.93 (0.11)	0.026*

*Expression *p* < 0.05.

In this paper, a Spearman rank correlation analysis was used to analyze the diversity index (Observed-outs, Shannon, Chao1) and blood routine parameters, and it was found that the MONO of AECOPD patients was negatively correlated with the alpha diversity of airway bacteria (Observed-outs, *r* = 0.505 ^**^), PCT was negatively correlated with the alpha diversity of bacteria (Shannon, *r* = −0.354*), D-D was negatively correlated with the alpha diversity of the microflora (Observed-outs, *r* = −0.372*), body temperature was negatively correlated with the alpha diversity of the microflora (Observed-outs, *r* = −0.540^**^), and HGB was negatively correlated with the alpha diversity of the flora (Observed-outs, *r* = −0.398*) (Table 3).

**TABLE 3:** Correlation analysis of alpha diversity of the airway microflora and clinical indicators in acute exacerbation of chronic obstructive pulmonary disease (AECOPD) group (R correlation coefficient).

	Observed-OUTs	Shannon	Simpson
Age	–0.150	–0.181	–0.106
Disease course	–0.005	0.223	0.067
WBC	–0.320	0.115	0.069
NEUT	–0.186	0.063	0.071
NEUT%	–0.010	0.044	0.110
LYM	–0.044	0.013	–0.072
LYM%	0.157	–0.120	–0.189
MONO	−0.505**	0.075	0.094
MONO%	–0.150	0.091	0.075
EO	–0.036	–0.177	0.097
EO%	–0.093	–0.125	0.033
BASO	–0.125	0.125	0.150
BASO%	–0.150	–0.090	–0.097
HGB	−0.398*	0.205	0.145
PLT	0.021	–0.256	0.145
hs-CRP	–0.283	–0.079	–0.130
PCT	0.181	−0.354*	–0.292
PA	0.184	0.211	0.183
ALB	0.128	–0.099	0.055
D-D	−0.372*	0.116	0.108
Length of stay	–0.077	0.222	0.102
Body temperature	−0.540**	–0.038	0.018

**Expression *p* < 0.01, *expression *p* < 0.05.

## Discussion

Although lung tissue is currently considered the preferred specimen for studying airway microecology, lung tissue specimens are difficult to obtain, and studies have found that upper airway and lower airway microflora are highly homologous, and there is no significant difference between the two species, except that the upper airway microflora is larger than the lower airway microflora (5). In addition, studies have shown that there is no significant difference in the composition of bacterial communities between spontaneous sputum samples and induced sputum samples (6). Thus, sputum was selected as the research sample in this study, and 16S rDNA high-throughput sequencing was performed to analyze and compare the airway microecology characteristics of AECOPD patients and healthy volunteers in Inner Mongolia. The correlation between the airway microecological diversity and clinical indicators, such as blood routine parameters, CRP, and PCT in patients with AECOPD was analyzed.

In our study, the airway flora structure of healthy volunteers in Inner Mongolia was mainly composed of Firmicutes, Proteobacteria, and Bacteroidetes, and the most common genera were Streptococcus, Neisseria, and Prevosia-7, which were roughly in line with reports from Inner Mongolia and other countries (7–9); however, there were slight differences in each study. For example, Streptococcus, Prevotella, and Veillonella were the main factors in a multi-center study on the airway microecology of healthy people in the United States (8). In China, a study from Southern Medical University (9) showed that, at the genus level in the lower respiratory tract of healthy people, Streptococcus was the most numerous, followed by Prevotella and Neisseria. Considering the differences in the living environment, eating habits, genetics, and other aspects of the study subjects, these factors can be expected to have a certain impact on airway microecology (10), so the results may be different. For example, smoking will lead to a significant increase in the abundance of Streptococcus, and a decrease in common symbiotic bacteria (11). However, studies on the effects of race, climate, and diet on respiratory tract flora are still in the initial stage, and more in-depth studies are needed.

In this experiment, Firmicutes and Proteobacteria were the main airway flora levels of AECOPD patients in Inner Mongolia. At the genus level, Streptococcus, Burkholderia, Veillonella, Actinobacteria, and Rothia are common, and LEfSe was used to analyze the difference in airway flora between healthy people and AECOPD patients. This analysis revealed that at the phylum level, the relative abundance of Bacteroidetes in the healthy group was higher; the relative abundance of pathogenic bacteria, such as Pseudomonas and Burkholderia, in the AECOPD group, was higher than that in the healthy group, and most of the genera that showed increased abundance in the AECOPD group belonged to Proteobacteria, which is in line with most previous reports (12, 13). However, there were differences. For example, in the study of Haldar et al. (13) the healthy population was dominated by Firmicutes and Bacteroidetes with relatively few Proteobacteria, while Proteobacteria were significantly increased in the COPD population and become the most important phylum. This may be due to factors such as age and smoking status among the COPD patients in each study. Studies have shown that the airway microbiome of smokers has a higher load of pathogenic bacteria (e.g., Streptococcus pneumoniae, Haemophilus influenzae, and Pseudomonas aeruginosa) (14). However, no matter how these bacterial groups change, they generally change in the direction of increasing pathogenic bacteria and decreasing symbiotic bacteria. Commensal bacterial changes may be equally important in the development of COPD. Some people think that it may be because co-bacteria can—to a certain extent—inhibit the growth of potential pathogens belonging to the same genus or family (15). This may be with symbiotic bacteria and these potential pathogenic bacteria competing for colonization area and nutrients, and some studies have found that some bacteria can produce active substances, such as antimicrobial peptides to inhibit or kill other flora (16). Similarly to our study, Wang et al. (17) also highlighted changes in non-pathogenic Proteobacteria in addition to the pathogenic microbiota during exacerbations, and it was found that Staphylococcus (potentially pathogenic bacteria) in the sputum of COPD patients and the absence of Veillonella, a potential commensal bacteria, were strongly associated with an increased risk of death after 1 year (18). This perspective highlights the importance of microbe-microbe interactions in maintaining homeostasis.

Our study showed that the alpha diversity of the airway microbiome in AECOPD patients was lower than that in healthy individuals, which is consistent with previous studies (6). This may be related to the proliferation of some pathogenic bacteria, which causes them to become dominant flora during acute exacerbations, inhibiting the growth of other flora, and resulting in a decline in flora diversity. Some studies have found that the decrease in diversity is related to the severity of the disease, and the microecology of patients with severe and advanced COPD was found to be lower than that of patients with moderate and severe disease (19). A study of the lung microbiome of COPD patients and a 1-year follow-up of the patients found that 1-year mortality increased significantly with a decrease in alpha diversity (18). These results suggest that airway microbial changes may be related to the exacerbation and prognosis of COPD.

A study showed that the airway microecology of patients with COPD could be significantly changed after 12 weeks of using ICS and or LABA preparations (20). Among the 34 patients with AECOPD included in this study, 17 of them used ICS + LABA on a regular basis. The medical history of preparations was more than half a year. To further study the effects of ICS and LABA on airway microecology, we analyzed the relationship between the diversity of airway flora in the AECOPD population and the use of ICS + LABA preparations. We found that the long-term inhalation of ICS + LABA preparations and alpha diversity was lower in AECOPD patients, and was associated with a relative reduction in Actinobacter and Haemophilus. Studies from Inner Mongolia and other countries have previously investigated the effects of hormones and long-acting β2 receptor agonists on the airway microecology, and also found that the treatment of COPD ICS can reduce the alpha diversity of the airway microecology (17, 21), but there is currently a lack of research on whether hormones can directly affect bacteria. However, studies have shown that, through indirect effects on the host, fluticasone, and budesonide inhibit the presence of *Haemophilus influenzae* in the airways (22), which is similar to our results. These data suggest that ICS may affect changes in the microbiome by promoting the growth of some bacteria and inhibiting others. However, there are few studies on the effect of β2 receptor agonists on airway microecology. At present, most clinical patients use a mixed preparation of ICS + LABA, which also makes it difficult to distinguish the specific effects of the two agents. Therefore, we need to better understand the effects of ICS, LABA, and other drugs on airway microecology, which may facilitate the more targeted use of these drugs.

This experiment found that PCT, MONO, D-dimer, body temperature, and airway microecological alpha diversity were negatively correlated. The elevation of these indicators all reflected the degree of inflammation in AECOPD, suggesting that the decrease in airway diversity may be associated with a more severe inflammatory response. This may be because the decline in the diversity of airway flora is due to the growth of pathogenic bacteria inhibiting the reproduction of other flora. Endotoxins and other metabolites produced by the mass reproduction of this pathogen can directly or indirectly affect the body’s inflammatory response (23). Studies have shown that the flagellin of *Pseudomonas aeruginosa* can induce bronchial epithelial cells to produce interleukin-6 (IL-6) and interleukin-8 (IL-8) by affecting the signal transduction pathway, aggravating the inflammatory response. The underlying mechanism by which *Pseudomonas aeruginosa* causes acute exacerbations of COPD has also been explained (24). We also found that hemoglobin content in AECOPD patients was also negatively correlated with airway alpha diversity. Decreased airway diversity has been previously shown to be associated with more severe airflow obstruction (6, 25), and severe hypoxemia can lead to secondary polycythemia (26). These results suggest that the decrease in airway microbial diversity may be related to the level of inflammation and severity of the disease.

## Conclusion

This study shows that the airway microecology of the AECOPD population in Inner Mongolia is different from that of the healthy population, and it is associated with drug use and some clinical indicators, which indicates that the airway microecological changes may be closely related to the acute attack of COPD. provides new ideas for diagnosis and treatment. There are many factors that affect airway microecology in the stable and acute stages of COPD. At present, this experiment has carried out part of the research on airway microecology in the acute exacerbation of COPD. In the next step, we need to use technologies such as metagenomics and metabolomics. The airway microecology (bacteria, fungi, viruses, etc.) of COPD patients with different stages and grades is analyzed by multi-factor, multi-center, and large samples to increase the influence of research results on the clinical diagnosis, treatment, and prognosis of COPD. Practical application.

## Data availability statement

The datasets presented in this study can be found in online repositories. The name of the repository and accession number can be found below: China National GeneBank (CNGB) Sequence Archive (CNSA), https://db.cngb.org/cnsa/, CNP0002927.

## Ethics statement

The study was reviewed and approved by the Ethics Committee of Inner Mongolia Medical University Institutional Review Board (approval No. YKD2018170). The patients/participants provided their written informed consent to participate in this study.

## Author contributions

S-FZ and X-XW designed and performed the experiments. YG, P-FL, J-RW, ML, and C-WL collected the data and performed the data analysis. X-ZY and S-WL designed the experiments and wrote the manuscript. All authors contributed to the article and approved the submitted version.
